# Comparison of Clinical, Laboratory and Immune Characteristics of the Monophasic and Biphasic Course of Tick-Borne Encephalitis

**DOI:** 10.3390/microorganisms9040796

**Published:** 2021-04-10

**Authors:** Petra Bogovič, Stanka Lotrič-Furlan, Tatjana Avšič-Županc, Miša Korva, Andrej Kastrin, Lara Lusa, Klemen Strle, Franc Strle

**Affiliations:** 1 Department of Infectious Diseases, University Medical Centre Ljubljana, Japljeva 2, 1525 Ljubljana, Slovenia; stanka.lotric-furlan@mf.uni-lj.si (S.L.-F.); franc.strle@kclj.si (F.S.); 2 Faculty of Medicine, University of Ljubljana, Vrazov trg 2, 1000 Ljubljana, Slovenia; 3 Faculty of Medicine, Institute for Microbiology and Immunology, University of Ljubljana, Zaloška 4, 1000 Ljubljana, Slovenia; tatjana.avsic@mf.uni-lj.si (T.A.-Ž.); Misa.Korva@mf.uni-lj.si (M.K.); 4 Faculty of Medicine, Institute for Biostatistics and Medical Informatics, University of Ljubljana, Vrazov trg 2, 1000 Ljubljana, Slovenia; andrej.kastrin@mf.uni-lj.si (A.K.); lara.lusa@mf.uni-lj.si (L.L.); 5 Department of Mathematics, Faculty of Mathematics, Natural Sciences and Information Technologies, University of Primorska, Glagoljaška 8, 6000 Koper, Slovenia; 6 Laboratory of Microbial Pathogenesis and Immunology, Division of Infectious Diseases, Wadsworth Center, New York State Department of Health, 120 New Scotland Ave, Albany, NY 12208, USA; klemen.strle@health.ny.gov

**Keywords:** tick-borne encephalitis, monophasic course, biphasic course, long-term outcome, cerebrospinal fluid, cytokines, chemokines, innate immune responses, adaptive immune responses

## Abstract

The biphasic course of tick-borne encephalitis (TBE) is well described, but information on the monophasic course is limited. We assessed and compared the clinical presentation, laboratory findings, and immune responses in 705 adult TBE patients: 283 with monophasic and 422 with biphasic course. Patients with the monophasic course were significantly (*p* ≤ 0.002) older (57 vs. 50 years), more often vaccinated against TBE (7.4% vs. 0.9%), more often had comorbidities (52% vs. 37%), and were more often treated in the intensive care unit (12.4% vs. 5.2%). Multivariate logistic regression found strong association between the monophasic TBE course and previous TBE vaccination (OR = 18.45), presence of underlying illness (OR = 1.85), duration of neurologic involvement before cerebrospinal fluid (CSF) examination (OR = 1.39), and patients’ age (OR = 1.02). Furthermore, patients with monophasic TBE had higher CSF levels of immune mediators associated with innate and adaptive (Th1 and B-cell) immune responses, and they had more pronounced disruption of the blood–brain barrier. However, the long-term outcome 2–7 years after TBE was comparable. In summary, the monophasic course is a frequent and distinct presentation of TBE that is associated with more difficult disease course and higher levels of inflammatory mediators in CSF than the biphasic course; however, the long-term outcome is similar.

## 1. Introduction

Tick-borne encephalitis (TBE) is an infection of the central nervous system. It is endemic in many parts of Asia and Europe. During the past few decades, the endemic regions have been expanding, and in almost all of the regions, the incidence has increased [1,2,3,4]. The disease is caused by at least three subtypes of tick-borne encephalitis virus (TBEV); European, Siberian, and Far-Eastern. Although the three subtypes are genetically and antigenically closely related, the clinical presentation of the disease caused by individual subtypes somewhat varies. Transmission of the infection to humans is predominantly through *Ixodes* spp. tick bites, and less often through the ingestion of unpasteurized (usually goat) milk or milk products. No etiologic treatment for TBE is available but safe and effective vaccines exist [2,5,6,7].

TBE caused by the European TBEV subtype usually has a biphasic course, in which neurologic involvement is preceded by an unspecific febrile illness. The initial (viremic) phase manifests with fever, headache, muscle pain, and fatigue, and usually lasts less than one week. After an improvement or even an asymptomatic interval of a few days, the second phase occurs, and presents as meningitis, meningoencephalitis, or meningoencephalomyelitis in 50%, 40%, and 5–10% of adult patients, respectively [2,6]. In Central Europe, where the disease is presumably caused by the European TBEV subtype, the fatality rate in adult patients is approximately 0.5%, around 5% of patients have permanent paresis, and at least one-third suffer from postencephalitic syndrome [8].

While in general, clinical manifestations of TBE are well defined, much less is known about individual aspects of the disease and their association with the course and outcome of TBE. In comparison to the biphasic course of TBE, which is common and well described, data on the monophasic course of the disease are incomplete. No direct comparison of patients with monophasic and biphasic course of TBE has been reported.

The aim of the present study was to appraise clinical, laboratory, and immune factors associated with the monophasic course of TBE, and to compare them between patients with monophasic and biphasic course of illness, with the intention to expand clinical and laboratory findings and to gain further insight into the pathogenesis of the disease.

## 2. Patients and Methods

### 2.1. Ethics

The study was carried out in concordance with the Declaration of Helsinki and was approved by the Medical Ethics Committee of the Republic of Slovenia (No 178/2/13, No 152/06/13, No 37/12/13). Each participant provided written informed consent.

### 2.2. Patients

The present study is based on 717 patients aged ≥18 years, who were hospitalized for TBE at the Department of Infectious Diseases, University Medical Centre Ljubljana, Slovenia in the period 2007–2012, assessed for long-term outcome in 2014, and enrolled in the study on the course and outcome of this disease. Clinical presentation of TBE and its long-term outcome have been reported previously [8,9] as has been the assessment of cytokine/chemokine responses during acute illness and follow-up visits in a small subset of these patients [10,11].

For 12 out of 717 patients, no reliable information on the monophasic or biphasic course of the disease was available. Consequently, the present study encompassed 705 adult patients with TBE, in whom detailed clinical and laboratory characteristics of the acute illness were appraised and compared according to monophasic (283 patients) or biphasic course (422 patients) of the illness, and a subset of 412 patients in whom the corresponding association with the long-term outcome of TBE was assessed. To gain an insight into the pathogenesis of TBE, cytokine/chemokine levels in matched serum and cerebrospinal fluid (CSF) samples, obtained early after the onset of neurologic involvement, were assessed and compared between 35 patients with the monophasic and 46 patients with the biphasic course of the disease. None of these 81 had been vaccinated against TBE.

### 2.3. Definitions

#### 2.3.1. Definition of Tick-Borne Encephalitis

TBE was defined with clinical symptoms/signs of central nervous system involvement, >5 × 10^6^ leukocytes/L in CSF, and the presence of serum IgM and IgG antibodies to TBEV [8,9,10,11].

#### 2.3.2. Meningitis, Meningoencephalitis, Meningoencephalomyelitis

Patients were classified as having: (a) meningitis when they had only symptoms/signs of meningeal inflammation (fever, headache, nausea, vomiting, rigidity of the neck); (b) meningoencephalitis when they had symptoms/signs indicating brain tissue damage (such as impaired consciousness, cognitive and/or concentration disturbances, tremor of extremities, tongue fasciculations, seizures) in addition to the findings of meningitis; or (c) meningoencephalomyelitis when they also had flaccid paresis [8,9,10,11].

#### 2.3.3. Biphasic and Monophasic Course

TBE was interpreted to have a biphasic course when neurologic involvement was preceded by an unspecific febrile illness and improvement with the duration of at least 1 day and up to 1 month. Patients who had no initial phase of the disease and present directly with central nervous system involvement were interpreted to have monophasic course of the disease.

#### 2.3.4. Tick-Borne Encephalitis-Associated Symptoms

During hospitalization and at each follow-up visit, patients were asked about the presence of subjective symptoms. Symptoms that newly developed or worsened since the onset of TBE, and which had no other known medical explanation, were interpreted as TBE-associated symptoms [8].

#### 2.3.5. Severity of Tick-Borne Encephalitis

Severity of the disease was categorized from least to most severe according to clinical diagnosis (meningitis, meningoencephalitis, meningoencephalomyelitis). In 460 patients (in all patients from 2009 on), the severity of TBE was also evaluated quantitatively using a standardized questionnaire, as reported previously: The presence, intensity, and duration of an individual symptom or sign of TBE were scored on a scale of 1–9 and the absence of a particular symptom or sign as zero; the severity score was defined as the sum of points. In a previous evaluation of this approach, the score ranges 0–8, 9–22, and >22 corresponded to clinically mild (meningitis), moderate, and severe disease, respectively [12].

#### 2.3.6. Post-Encephalitic Syndrome (PES)

PES was defined with the presence of ≥2 subjective symptoms fulfilling criteria for TBE-associated symptoms or with ≥1 objective neurological sign at the last follow-up visit 2–7 years after TBE [8].

### 2.4. Tick-Borne Encephalitis Virus Antibody Determination

Antibodies to TBEV were measured using the Enzygnost^®^ Anti-TBE Virus (IgM, IgG) test (SiemensGmbH, Marburg, Germany) according to the manufacturer’s protocol.

### 2.5. Chemokine and Cytokine Determinations

The levels of 24 cytokines/chemokines associated with innate (GM-CSF, IFNα, IL-10, IL-1β, IL-6, IL-8, TNF, CCL2, CCL3) and adaptive Th1 (IFNγ, IL-12p40, IL-12p70, CXCL10, CXCL9, CCL19), Th17 (IL-17F, IL-17A, IL-22, IL-21, IL-23, IL-17E, IL-27), and B cell immune response (CXCL12, CXCL13) were assessed in matched CSF and serum samples obtained at first visit during meningoencephalitic phase of TBE using bead-based multiplex assays (Luminex, EMD Millipore Burlington, MA, USA). To minimize inter-assay variation, all measurements were performed on the same day in one complete experiment.

### 2.6. Statistical Analyses

Continuous variables were summarized with median values and interquartile ranges (IQRs), and discrete variables were summarized with counts and percentages. All percentages were reported with 95% confidence intervals (CIs). Comparisons between groups (i.e., monophasic vs. biphasic disease progression) were based on a Wilcoxon rank-sum test for continuous variables and Fisher’s exact test for discrete variables. All *p*-values < 0.05 were considered statistically significant. The Benjamini and Hochberg (BH) procedure to correct for false positives due to multiple comparisons was used to adjust *p*-values where appropriate.

Both univariate and multiple regressions were used to assess the association between a disease outcome and 13 preselected clinical and laboratory covariates. Covariates included in the logistic models were selected by expert opinion (P.B. and F.S.) and were decided upon without knowledge of the observed outcome. Continuous covariates were standardized according to IQRs. Patients with biphasic disease course were defined as the reference group in all logistic models. Regression models were reported with odds ratios (ORs) and 95% confidence intervals. Corresponding *p*-values were calculated using the Wald test. CSF and serum concentrations of cytokines/chemokines in patients with monophasic and biphasic course of TBE were compared using the Wilcoxon rank-sum tests. All statistical analyses were performed using R software [13].

## 3. Results

**Figure 1:**
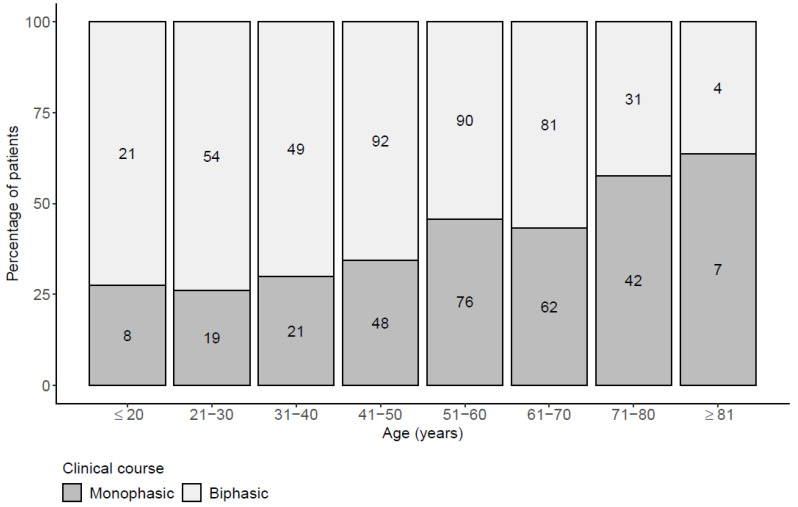
Proportion of patients with monophasic and biphasic course of tick-borne encephalitis according to age. The raw number of patients in the two groups is shown separately in each bar.

Of 705 patients who qualified for the study, 283 (40.1%) had monophasic and 422 (59.9%) had biphasic course of illness. Comparison of the two groups revealed that patients with monophasic course were statistically significantly older (57 vs. 50 years), more often had underlying diseases (52% vs. 37%), were more often vaccinated against TBE (7.4% vs. 0.9%), had longer duration of illness (neurologic involvement) prior to admission to hospital and CSF examination (5 vs. 4 days), and had more pronounced disruption of blood brain barrier as indicated by higher albumin as well as IgG quotient. Detailed findings are summarized in Table 1. The proportions of patients with monophasic course of TBE stratified according to age are depicted in Figure 1. In addition, patients with the monophasic course of the illness more often had more severe disease as evidenced by the higher proportion of monophasic course in patients with meningoencephalitis (182/439, 41.5%) or meningoencephalomyelitis (20/39, 51.3%) than in patients with meningitis (81/227, 35.7%), and by higher severity score, although the differences were not statistically significant. Moreover, significantly more patients with the monophasic course needed treatment in the intensive care unit (35/283, 12.4% with monophasic course versus 22/422, 5.2% with biphasic course; *p* = 0.001).

Univariate logistic regression analyses confirmed these findings. Several factors were significantly associated with monophasic course of TBE, including age of patients (OR = 1.02), the presence of underlying illness (OR = 1.87), previous vaccination against TBE (OR = 8.38), duration of neurologic involvement before CSF examination (OR = 1.34), severity of illness according to severity score (OR = 1.02), albumin quotient (OR = 1.58), IgG quotient (OR = 1.48), and IgG antibody levels against TBEV in serum (OR = 1.19). Since these individual factors may be related, we also performed multiple regression analyses. Several findings remained statistically significantly associated with monophasic course of TBE using multivariate testing, including: Age of patients (OR = 1.02; 95% CI: 1.00–1.05), the presence of underlying illness (OR = 1.85; 95% CI: 1.04–3.30), previous vaccination against TBE (OR = 18.45; 95% CI: 1.73–367.10), and duration of neurologic involvement before CSF examination (OR = 1.39; 95% CI: 1.25–1.56) while other covariates (severity of illness, albumin, and IgG quotient, IgG antibody levels against TBEV in serum) did not (Table 2).

The outcome 2–7 years after TBE was available for 412 patients. In contrast to several distinctions in the early course of TBE, the long-term outcome of the disease revealed no significant differences comparing patients with the monophasic and biphasic courses of illness. PES was present in 56/174 (32.2%) patients with the monophasic course of the disease and in 81/238 (34.0%) patients with biphasic TBE (*p* = 0.774). There was also no statistically significant difference comparing the two groups according to the subset of patients with PES, represented by the presence of at least one objective finding with or without subjective symptoms, which was found in 10/174 (5.7%) patients with the monophasic course of the disease and in 8/238 (3.4%) patients with the biphasic TBE (*p* = 0.354).

In addition to the laboratory and clinical parameters, cytokine/chemokine levels were assessed in matched serum and CSF samples obtained early after the onset of neurologic involvement and compared between 35 patients with monophasic and 46 patients with biphasic course of the disease. The comparison revealed several differences in the cytokine/chemokine levels in CSF (Table 3). In patients with the monophasic course, the CSF levels of all analyzed cytokines/chemokines were either higher or equal to, but never lower than, the levels found in patients with biphasic course of TBE. Specifically, CSF levels of all tested cytokines/chemokines representing innate immunity (9/9), adopted Th1 immune response (6/6), and B cell immune response (2/2) were higher in patients with monophasic that in patients with biphasic course, while the levels of Th17 immune mediators were identical for all but one mediator (6/7). The differences were most pronounced (*p* < 0.05) for GM-CSF, IL-1β, TNFα, and IL-10 (representing innate immunity); IFNγ and CXCL9 (representing Th1 immune response); and B-cell immune mediator CXCL13. In contrast to the findings in CSF, very few distinctions were observed in serum, except that the levels of CXCL13 in serum were lower in patients with the monophasic course of illness than in patients with the biphasic course. Nevertheless, after adjustment for multiple comparisons these findings were not statistically significant.

## 4. Discussion

Although TBE has been studied for more than 90 years [14] and the disease has been well described, several details remain uncertain. In particular, little is known about the monophasic course of the disease, including its frequency, factors associated with such course as well as its (long-term) outcome. Furthermore, little is known about immune mediators in TBE; the information is predominantly limited to description of the levels of the cytokines/chemokines in the meningoencephalitic phase of TBE and the attempts to assess the levels in relation to severity of illness and to favorable or unfavorable outcome [10,11,15,16,17,18,19,20]. In the present study, we performed a detailed analysis of the clinical and laboratory findings in a large group of patients with TBE according to the monophasic or biphasic course of illness, compared the long-term outcome of the two presentations, and tried to get insight into the levels of immune mediators in matched serum and CSF samples obtained from subsets of patients from each of the two subgroups.

In the majority of patients with TBE (presumably) caused by the European TBEV subtype, neurologic involvement appears after an initial unspecific febrile illness and a short improvement that is labeled as the biphasic course. Nonetheless, some patients with TBE have no (obvious) initial phase of the disease and present directly with central nervous system involvement. According to the reports consisting of 80 or more cases of TBE (presumably) caused by the European TBEV subtype, such a monophasic course is present in 13–44% of patients [21,22,23,24,25,26,27,28,29,30]. In the present study, 283/705 patients (40.1%) had a monophasic course of the disease, which is a rather high proportion. Our patients did not differ substantially from those in other European reports according to the majority of parameters listed in Table 1. However, in our study, the predominant form of acute illness was meningoencephalitis (62.1%), unlike most other series where meningoencephalitis represented <50%, and together with meningoencephalomyelitis <55% of adult patients [22,23,26,28,31].

One of the main aims of the present study was to analyze clinical and laboratory characteristics associated with the monophasic course of TBE. Some studies showed that patients with monophasic presentation of TBE have a more severe clinical course of the disease than those with biphasic course [23,25,29,32] while several others did not find such association. In addition, some reports on patients with severe TBE who needed intensive care management [33,34] show an unusually high proportion of those with monophasic course (15/31, 48.4% and 21/33, 63.6%, respectively) suggesting that a more severe course of TBE is associated with the monophasic course of the disease. The present study revealed that in comparison to patients with the biphasic course those with the monophasic course more often present with more severe illness (meningoencephalitis, meningoencephalomyelitis), have higher severity score, and more often needed treatment in intensive care unit (ICU). However, of these parameters, the difference was statistically significant only for ICU treatment: Of 57 patients treated in the ICU, 35 (61.4%) had the monophasic and 22 (38.6%) had the biphasic course. The high proportion of the monophasic course is in good concord with previous reports on TBE patients treated in the ICU [33,34].

The monophasic course of illness has also been reported for patients who develop TBE despite being vaccinated for this disease [35,36]. In one of the reports, 27/39 (69.2%; 95% CI: 52.4–83.0) previously vaccinated patient had a monophasic course of the disease while in sex- and age-matched unvaccinated patients with TBE, the ratio was 31/78 (39.7%; 95% CI: 28.8–51.5) (*p* = 0.006; adjusted *p* = 0.020) [35]. In the other report, 41/53 (77.4%; 95% CI: 63.8–87.7) previously vaccinated patients had the monophasic course of illness [36]. Consistent with these reports, in the present study, we found a strong association of the monophasic course of TBE with previous vaccination against this disease: 84% of previously vaccinated patients had the monophasic course of illness versus 38.5% of unvaccinated patients; in univariate and multivariate analysis, ORs for monophasic TBE in previously vaccinated persons were 8.4 and 18.5, respectively.

In the present study, direct comparison as well as univariate analyses also showed that patients with the monophasic course of TBE were older and had a higher proportion of underlying diseases than those with the biphasic course of the disease, and that they had longer duration of illness prior to diagnosis and more pronounced disruption of the blood–brain barrier as indicated with higher albumin and IgG indexes, all of which could contribute to greater disease severity. A speculative explanation for age difference would be that the monophasic course of TBE is associated with older age because elderly persons less often develop pronounced fever and consequently have higher chances to overlook the initial phase of illness (which is, as a rule, milder than the phase associated with neurologic involvement) and/or to recall a recent prior illness. Yet, even if such explanation might have been partly valid for the oldest patients, it is not applicable to the younger age groups. Several other factors could directly or indirectly contribute to the association of monophasic course with older age, including higher frequency of underlying illnesses, later decision to seek for medical help in older than in younger patients, and—due to consequent longer duration of illness—more pronounced disruption of the blood–brain barrier. Indeed, in multiple logistic regression analyses, albumin and IgG quotient and IgG antibody levels against TBEV in serum were not found to be statistically significantly associated with the monophasic course of TBE. However, the age of the patients, the presence of underlying illness, previous vaccination against TBE, and the duration of neurologic involvement before CSF examination remained statistically significantly associated, also using multivariate testing, suggesting that these factors were independently associated with the monophasic course of illness. We do not have an explanation for the association of the presence of underlying illness and previous vaccination against TBE with a monophasic course of TBE. A possible explanation for the longer duration of illness prior to CSF examination and diagnosis is that in Slovenia, TBE is perceived as a two-phase illness and thus the awareness and recognition of TBE in the general population and presumably among medical doctors is higher with the biphasic than the monophasic course of illness.

In contrast to differences between monophasic and biphasic courses in the acute phase of illness, no distinctions were found 2–7 years after TBE with regards to the frequency of PES (it was present in 32% of patients with monophasic course of the disease and in 34% of patients with biphasic TBE) as well as its severity.

To obtain insight into the immune mechanisms behind monophasic and biphasic courses of TBE, we assessed and compared cytokine/chemokine levels in matched serum and CSF samples obtained early after the onset of neurologic involvement. The comparison disclosed several differences in CSF levels but no substantial differences in serum levels of the mediators between the two groups. To help interpret the results, we grouped the cytokines and chemokines according to their main biological function. Although after adjustment for multiple comparisons none of the differences remained statistically significant, the most prominent finding was that all immune mediators representing innate immunity (9/9), Th1 immune response (6/6), and B-cell immune response (2/2) had higher CSF levels in monophasic than biphasic course of illness, while the levels of Th17 immune mediators were identical for all but one mediator (6/7) (Table 3). Since cytokine/chemokine levels were determined in only a subset of patients (35 with monophasic and 46 with biphasic course) and the number of immune mediators tested was high (24), it is not surprising that no statistically significant associations were found after adjustment for multiple testing. However, the absence of statistically significant differences should not obscure the fact that CSF concentrations of all mediators representing innate, Th1, and B cell immune response were uniformly higher in monophasic than in biphasic course of illness. Indeed, the differences for several of these immune mediators were statistically significant before correction for multiple comparisons, implying that the findings are likely biologically relevant. Nevertheless, this is one of the main limitations of the study and will need to be validated in a larger cohort of patients. Moreover, all patients were seen at a single medical center, and while the results may be valid for Central Europe where the disease is caused by the European subtype of TBEV, they may not be representative of other regions where different TBEV subtypes prevail.

## 5. Conclusions

In the present study of 705 adult patients with TBE, 40% had a monophasic course of TBE. Compared to patients with a biphasic course, those with a monophasic course were older and they more often had comorbidities, vaccination against TBE, and longer duration of neurologic involvement before CSF examination. Moreover, they had higher CSF levels of immune mediators representing innate and Th1 and B-cell immune responses and were more often admitted to the ICU, indicative of more severe disease. Although the reasons for these associations are not yet know and require further investigation, we think these findings may hold practical value for patients and their physicians by raising awareness and recognition of the high frequency of monophasic TBE course and the associated disease severity.

## Figures and Tables

**Table 1:** Factors associated with monophasic course of tick-borne encephalitis.

Factors	All Patients	Course of Illness		Adjusted *p*-Value
		Biphasic	Monophasic	
	705	422	283	
Female sex	305 (43.3)	178 (42.2)	127 (44.9)	0.565
Age	54 (41–64)	50 (37.2–61)	57 (45–67)	**<0.001**
Underlying illness	304 (43.1)	156 (37.0)	148 (52.3)	**<0.001**
Vaccinated against TBE	25 (3.5)	4 (0.9)	21 (7.4)	**<0.001**
Duration of neurologic involvement before CSF examination (days) ^a^	4 (3–6)	4 (3–5)	5 (4–7)	**<0.001**
Clinical diagnosis				0.225
meningitis	227 (32.2)	146 (34.6)	81 (28.6)	
meningoencephalitis	439 (62.3)	257 (60.9)	182 (64.3)	
meningoencephalomyelitis	39 (5.5)	19 (4.5)	20 (7.1)	
Severity score ^b^	12 (5–18)	11 (5–17)	12 (5–21)	0.225
Treated in ICU	57 (8.1)	22 (5.2)	35 (12.4)	**0.002**
Death within the first month after the onset of TBE	3 (0.4)	2 (0.5)	1 (0.4)	>0.999
CSF leukocyte count (×10^6^/L)	86 (44–149.5)	86.5 (42–155)	86 (48–149.5)	0.996
CSF granulocyte count (×10^6^/L) ^c^	30 (11–57.8)	32 (12–59)	28 (11–54)	0.512
CSF lymphocyte count (×10^6^/L) ^c^	42.5 (17–87)	40 (16–91)	43 (20–85)	0.409
Albumin quotient ^d,e^	10.2 (7.9–13.9)	9.6 (7.6–12.6)	11.6 (8.6–15.3)	**<0.001**
IgG quotient ^d,e^	5.1 (4.0–6.8)	4.8 (3.9–6.6)	5.6 (4.3–7.9)	**<0.001**
IgG antibody levels against TBEV in serum (U/mL)	37.2 (19.6–63.1)	36.7 (18.6–61.9)	38.3 (20.7–66.2)	0.225

Data are medians (interquartile ranges) or number (%). For statistical comparison of numerical variables Man–Whitney test was used, comparison of categorical variables was performed with Fisher’s exact test. ^a^ Data missing for 53 patients (21 with monophasic and 32 with biphasic illness). ^b^ Data missing for 245 patients (96 with monophasic and 149 with biphasic illness). ^c^ Data missing for 3 patients (2 with monophasic and 1 with biphasic illness). ^d^ Data missing for 172 patients (78 with monophasic and 94 with biphasic illness). ^e^ Albumin (IgG) quotient is a quotient between CSF and serum albumin (IgG) concentration. TBE, tick-borne encephalitis; CSF, cerebrospinal fluid; ICU, intensive care unit; TBEV, tick-borne encephalitis virus.

**Table 2:** Factors associated with the monophasic course of tick-borne encephalitis and multiple logistic regression according to univariate.

Factors	Univariate		Multiple	
	OR (95% CI)	*p*-Value	OR (95% CI)	*p*-Value
Male sex	1.12 (0.82–1.51)	0.479	1.18 (0.70–1.98)	0.530
Age	1.02 (1.01–1.04)	**<0.001**	1.02 (1.00–1.05)	**0.021**
Underlying illness	1.87 (1.38–2.54)	**<0.001**	1.85 (1.04–3.30)	**0.035**
Vaccinated against TBE	8.38 (3.15–28.95)	**<0.001**	18.45 (1.73–367.10)	**0.029**
Duration of neurologic involvement before CSF examination (days) ^a^	1.34 (1.25–1.44)	**<0.001**	1.39 (1.25–1.56)	**<0.001**
Clinical diagnosis				
meningoencephalitis	1.28 (0.92–1.78)	0.149	0.50 (0.23–1.08)	0.081
meningoencephalomyelitis	1.90 (0.96–3.78)	0.067	0.34 (0.06–1.62)	0.185
Severity score ^b^	1.02(1.00–1.04)	**0.030**	1.03 (0.99–1.08)	0.146
CSF leukocyte count (×10^6^/L)	1.00 (1.00–1.00)	0.197	1.00 (0.97–1.03)	0.944
CSF granulocyte count (×10^6^/L) ^c^	1.00 (1.00–1.00)	0.464	1.00 (0.97–1.03)	0.887
CSF lymphocyte count (×10^6^/L) ^c^	1.00 (1.00–1.00)	0.239	1.00 (0.97–1.03)	0.908
Albumin quotient ^d,e^	1.58 (1.29–1.94)	**<0.001**	0.91 (0.46–1.79)	0.792
IgG quotient ^d,e^	1.48 (1.23–1.80)	**<0.001**	1.47 (0.75–2.95)	0.271
IgG antibody levels against TBEV in serum (U/mL)	1.19 (1.09–1.33)	**<0.001**	0.96 (0.78–1.20)	0.684

^a^ Data missing for 53 patients (21 with monophasic and 32 with biphasic illness) ^b^ Data missing for 245 patients (96 with monophasic and 149 with biphasic illness) ^c^ Data missing for 3 patients (2 with monophasic and 1 with biphasic illness) ^d^ Data missing for 172 patients (78 with monophasic and 94 with biphasic illness) ^e^ Albumin (IgG) quotient is a quotient between CSF and serum albumin (IgG) concentration. OR, odds ratio; CI, confidence interval; TBE, tick-borne encephalitis; CSF, cerebrospinal fluid; TBEV, tick-borne encephalitis virus.

**Table 3:** Cerebrospinal fluid (CSF) and serum concentrations of cytokines/chemokines obtained in patients during the early meningoencephalitic phase of tick-borne encephalitis: Comparison of findings in patients with monophasic and biphasic clinical course.

	CSF Concentrations (pg/mL) Median (IQR)				Serum Concentrations (pg/mL) Median (IQR)			
Cytokine/ Chemokine	Monophasic	Biphasic	*p*-Value	Adjusted *p*-Value	Monophasic	Biphasic	*p*-Value	Adjusted *p*-Value
**Innate**								
GM-CSF	2.45 (1.40)	1.68 (1.43)	0.026	0.085	1.01 (1.03)	0.79 (1.37)	0.837	0.985
IFNα	33.64 (23.59)	29.85 (25.52)	0.609	0.762	3.58 (9.98)	3.58 (14.19)	0.946	0.985
IL-1β	1.25 (1.29)	1.00 (0.65)	0.041	0.110	0.44 (0.45)	0.51 (0.94)	0.217	0.985
IL-6	243.41 (417.30)	145.05 (143.29)	0.288	0.411	0.30 (0.93)	0.30 (1.59)	0.591	0.985
IL-8	98.30 (96.35)	72.75 (58.35)	0.088	0.194	4.38 (33.20)	10.96 (23.05)	0.326	0.985
TNFα	4.57 (4.06)	2.78 (3.15)	0.007	0.073	12.31 (35.48)	11.86 (19.06)	0.985	0.985
CCL2	335.46 (313.94)	325.18 (444.32)	0.501	0.669	118.58 (122.42)	116.37 (91.95)	0.886	0.985
CCL3	11.95 (11.62)	10.93 (12.01)	0.954	0.954	1.01 (30.52)	1.01 (28.44)	0.897	0.985
IL-10 *	0.23 (0.00)	0.23 (0.00)	0.020	0.084	0.23 (0.00)	0.23 (0.00)	0.738	0.985
**Th1**								
IFNγ	43.42 (78.98)	21.99 (28.28)	0.011	0.075	1.19 (2.56)	1.19 (1.67)	0.616	0.985
IL-12p40 *	36.62 (50.12)	36.09 (41.49)	0.852	0.897	1.18 (0.00)	1.18 (0.00)	0.221	0.985
IL-12p70	0.72 (0.69)	0.54 (0.54)	0.044	0.110	0.38 (0.44)	0.38 (0.81)	0.953	0.985
CXCL9	49.97 (91.75)	25.06 (41.07)	0.021	0.084	19.51 (25.15)	14.75 (14.92)	0.451	0.985
CXCL10	8279.00 (9533.00)	5782.00 (6858.25)	0.160	0.261	96.87 (140.08)	84.97 (82.70)	0.213	0.985
CCL19	148.18 (200.78)	134.10 (146.83)	0.668	0.786	11.82 (14.14)	12.23 (15.17)	0.548	0.985
**Th17**								
IL-17F *	0.32 (0.00)	0.32 (0.00)	NaN	NaN	0.32 (0.00)	0.32 (0.00)	0.321	0.985
IL-17A *	0.31 (0.00)	0.31 (0.00)	NaN	NaN	0.31 (0.00)	0.31 (0.00)	0.971	0.985
IL-22 *	0.21 (0.00)	0.21 (0.00)	0.107	0.194	0.21 (0.17)	0.21 (0.27)	0.352	0.985
IL-21 *	2.71 (0.00)	2.71 (0.00)	0.107	0.194	7.59 (14.00)	5.70 (11.17)	0.525	0.985
IL-23 *	0.25 (0.00)	0.25 (0.00)	NaN	NaN	0.25 (0.00)	0.25 (0.00)	0.638	0.985
IL-17E *	0.32 (0.00)	0.32 (0.00)	NaN	NaN	0.32 (0.00)	0.32 (0.00)	0.047	0.561
IL-27	4.05 (6.12)	2.63 (3.77)	0.170	0.261	2.69 (1.96)	2.56 (2.21)	0.943	0.985
**B-cell**								
CXCL12	27.18 (26.90)	22.40 (29.39)	0.830	0.897	19.40 (14.61)	22.88 (13.92)	0.721	0.985
CXCL13	11.91 (39.66)	1.57 (15.23)	0.006	0.073	29.02 (24.97)	43.19 (31.46)	0.018	0.444

Note: To control for false positives, the raw *p*-values were adjusted using the Benjamini and Hochberg procedure. NaN values indicate that compared groups have the same distribution. * Medians and IQRs are the same but the other data are not.

## Data Availability

The data presented in this study are available on request from the corresponding author. The data are not publicly available due to protection of patient personal information.
